# Multi-Point Flexible Temperature Sensor Array and Thermoelectric Generator Made from Copper-Coated Textiles

**DOI:** 10.3390/s21113742

**Published:** 2021-05-28

**Authors:** Justus Landsiedel, Waleri Root, Noemí Aguiló-Aguayo, Heinz Duelli, Thomas Bechtold, Tung Pham

**Affiliations:** 1 Research Institute for Textile Chemistry and Textile Physics, University of Innsbruck, Hoechsterstrasse 73, 6850 Dornbirn, Austria; Justus.Landsiedel@uibk.ac.at (J.L.); textilchemie@uibk.ac.at (W.R.); noemi.aguilo-aguayo@uibk.ac.at (N.A.-A.); thomas.bechtold@uibk.ac.at (T.B.); 2 Fachhochschule Vorarlberg, CAMPUS V, Hoechsterstrasse 1, 6850 Dornbirn, Austria; heinz.duelli@fhv.at

**Keywords:** flexible, temperature sensor, thermocouple, energy harvesting

## Abstract

The integration of electrical functionality into flexible textile structures requires the development of new concepts for flexible conductive material. Conductive and flexible thin films can be generated on non-conductive textile materials by electroless metal deposition. By electroless copper deposition on lyocell-type cellulose fabrics, thin conductive layers with a thickness of approximately 260 nm were prepared. The total copper content of a textile fabric was analyzed to be 147 mg per g of fabric, so that the textile character of the material remains unchanged, which includes, for example, the flexibility and bendability. The flexible material could be used to manufacture a thermoelectric sensor array and generator. This approach enables the formation of a sensor textile with a large number of individual sensors and, at the same time, a reduction in the number of electrical connections, since the conductive textile serves as a common conductive line for all sensors. In combination with aluminum, thermoelectric coefficients of 3–4 µV/K were obtained, which are comparable with copper/aluminum foil and bulk material. Thermoelectric generators, consisting of six junctions using the same material combinations, led to electric output voltages of 0.4 mV for both setups at a temperature difference of 71 K. The results demonstrate the potential of electroless deposition for the production of thin-film-coated flexible textiles, and represent a key technology to achieve the direct integration of electrical sensors and conductors in non-conductive material.

## 1. Introduction

Electronic textiles (e-textiles) have attracted great interest in recent years, as the combination of electronics with textiles could herald a new era in mobile electronics and everyday devices, such as cell phones, sports aids, or the like. In this union, the rigid and highly functional world of electronics meets the lightness and flexibility of textiles. In order to make the connection between these two worlds successful, one system must be adapted to the other. In the case of e-textiles, this includes the development of flexible electronics, as well as the electrical functionalization of textiles to facilitate integration. Ideally, the electrical functionalization of textiles not only supports the construction of simple electronic components such as energy generators or sensors, but is also part of them. While there are a variety of principles of operation for sensors and power generators, thermocouples provide the best overlap of both [1,2]. Thermocouples can be used to either measure temperatures or generate energy based on temperature gradients in a closed circuit consisting of two dissimilar conductors [3]. Thermocouples can be constructed from various materials and material combinations, such as carbon fibers embedded in a polymer matrix [4], metals or alloys [5], metals in different thicknesses [6,7], or conductive polymers [3].

To achieve the electrical functionalization of textiles and the implementation of energy generators and sensors, there are the following two possible ways: (a) the incorporation of conductive yarns and threads into the body of textiles, or (b) the functionalization of constructed textiles by suitable printing and coating technologies. Each approach has its specific advantages and disadvantages, such as high conductivity paired with low flexibility when using electrically conductive wires and yarns, or vice versa when using conductive prints and coatings. In the electrical functionalization of textiles by means of printing and coating techniques, the most frequently used materials are (a) conductive polymers such as poly(3,4-ethylenedioxythiophene) polystyrene sulfonate PEDOT:PSS [8], and (b) metals such as copper or silver [9]. While the use of conductive polymers initially appears more appropriate due to the similarity in mechanical properties, metal coatings show advantages such as better electrical conductivity, higher abrasion resistance and better recyclability.

In this study, thermocouples are constructed from copper-coated fabrics and aluminum strips since both of these metals exhibit good conductivities, good recyclability, and are low-cost materials compared to conductive polymers [9,10]. The electrically conductive textiles were manufactured by the electroless deposition of copper—a process that enables the formation of thin copper films with resistance values of a few ohm/square on the entire surface of the substrate [11]. Compared to printing technologies that have been utilized for the manufacture of thermocouples in more recent publications, the partial coating of the fabrics will also avoid the loss of functionality due to abrasion of the deposited layers from the textile surface. Another particular advantage of thin-film metal coatings is expected to be their low thermal conductivity [12], which reduces the unwanted heat transfer along the flat textile structure.

This work focuses on the characterization of the morphology and the thermoelectric properties of copper–aluminum thermocouples in a flexible textile-based sensor array, and can be seen as a demonstrator and proof-of-concept for the construction of textile-integrated thermocouples. In a further approach, the use of an arrangement of three thermocouples connected in a series was tested as a thermoelectric generator for electrical energy.

## 2. Materials and Methods

### 2.1. Materials

A plain-woven 100% cellulose lyocell (CLY) fabric with a weight of 143 g/m^2^, linear density (λ_m_) of 1.3 dtex, 0.24 mm thickness and thread count of 4000/m in warp and 3200 in weft direction was used as textile substrate material, which was provided by Lenzing AG (Lenzing, Austria). Copper foil of 0.1 mm thickness was supplied by EMSURE (Darmstadt, Germany). The aluminum foil was supplied by RS components (Gmuend, Austria).

Tin(II) chloride dihydrate (SnCl_2_ 2H_2_O) and formaldehyde (CH_2_O, 36.5 wt.%) were supplied by Riedel-de-Haen (Seelze, Germany). Copper sulfate pentahydrate (CuSO_4_ 5H_2_O, 99.5%), embittered ethanol (C_2_H_6_O, >98%; MEK, 1%) and ammonia solution (NH_3_, 25 wt.%) were obtained from Carl ROTH GmbH & Co. KG (Karlsruhe, Germany). Sodium carbonate (Na_2_CO_3_, 99 wt.%) was supplied by MERCK (Darmstadt, Germany). Silver nitrate (AgNO_3_, 99.0 wt.%) was obtained from VWR PROLABOR (Leuven, Belgium). Sodium hydroxide (NaOH, 50 wt.% solution) was obtained from Deuring GmbH & Co. KG (Hörbranz, Austria). Potassium hydrogen-L-tartrate (C_4_H_5_KO_6_, 99 wt.%) was supplied by Fluka (Buchs, Switzerland). All chemicals were used without any further purification.

### 2.2. Manufacture of Copper-Coated Cellulose Fabrics

The process to manufacture the copper-coated cellulose lyocell fabrics involved the following three steps: (a) surface activation using tin chloride, (b) silver seeding, and (c) copper deposition. For surface activation, the lyocell fabric was dipped in a solution of 2.9 mmol SnCl_2_ in 100 mL ethanol. Afterwards the fabrics were dried for 1 h at room temperature. The seeding process was carried out by immersing the activated lyocell fabrics for 1 min in a solution of 4.2 mmol AgNO_3_, 26 mmol NH_3_ and 100 mL H_2_O (DI). Subsequently, the fabrics were rinsed with H_2_O (DI) to remove unbound excess amounts of silver from the surface, and were dried for 1 h at room temperature. For the deposition of copper, the silver seeded fabrics were immersed into a solution of 2.8 mmol CuSO_4_, 2.9 mmol Na_2_CO_3_, 7.9 mmol C_4_H_5_KO_6_, 24.7 mmol NaOH in 100 mL H_2_O (DI). The reaction was initiated by the addition of 36 mmol H_2_CO. The deposition was carried out for 1 h at ambient temperature. Afterwards, the fabrics were rinsed with an excess amount of H_2_O (DI) to remove unbound copper particles from the surface.

### 2.3. Microscopy and Material Characterization

The topology and structure of the copper-coated substrate were investigated using scanning electron microscopy (SEM) (JEOL JSM 7100F, JEOL, Tokyo, Japan), laser scanning microscopy (LSM) (VK X100, KEYENCE, Tokyo, Japan) and optical microscopy (Olympus SZX16, Olympus Europa SE & Co. KG, Hamburg, Germany). Cross-section cuts were made using ultramicrotomy (EM UC7, Leica, Vienna, Austria). The SEM measurements were performed without deposition of any additional materials using working distances between 4.8 mm and 5.9 mm and accelerating voltages between 10 and 20 keV. The elemental composition of the copper-coated textiles was analyzed using energy dispersion X-ray spectroscopy (EDX) (JEOL JSM 7100F, JEOL, Tokyo, Japan).

The determination of copper content was carried out with atomic absorption spectroscopy (AAS) according to DIN EN ISO 17294-2 and DIN 38406-7 on F-AAS (contrAA 300, Analytik Jena GmbH, Jena, Germany) with 100-mm burner and air acetylene flame.

### 2.4. Determination of Temperature Sensitivity and Thermoelectric Energy

In order to determine the sensitivity of the thermocouple made of copper-coated textiles and aluminum strips, a beaker filled with hot water was placed on the measuring terminals. The actual temperature of the liquid in the beaker and the associated output voltage response were recorded over time using a voltmeter and a laboratory electric thermometer until ambient temperature was reached. The temperatures described are absolute temperatures, no temperature differences. The thermoelectric energy that develops between a heated junction and a cold junction was determined using the same method.

### 2.5. Thermoelectric Generator

A combination of aluminum strips (Advance Tapes AT521, 10 mm width, RS Components) with copper-coated textile strips (7 cm × 1 cm) was chosen to manufacture a thermoelectric generator. As described above, three thermocouples were connected in a series. For reference experiments a copper strips were used instead of the copper-coated textile material. The temperature difference between the material connections was measured with a digital thermometer (K-Type sensors, Keithley Instruments, Solon, OH, USA). A temperature difference of 71 K was realized by placing a hot-water filled beaker on one series of junctions and by cooling the other series with pre-chilled cooling thermal pack. The internal resistance and the thermoelectric output were measured for 70 s with a potentiometer (BioLogic VSP-3e, Seyssinet-Pariset, France).

## 3. Results and Discussion

### 3.1. Manufacture of Textile-Based Thermocouples

**Figure 1:**
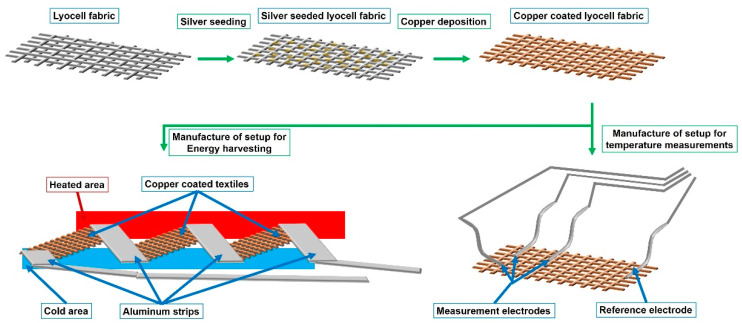
Manufacture of textile-based thermocouples usable for temperature measurements and energy harvesting, made from copper-coated lyocell fabrics.

Electroless copper deposition was used to form a thin layer of copper as a conductive coating on a non-conductive lyocell-type cellulose fabric. After the formation of Ag seeds, the reductive deposition of Cu was initiated in an alkaline solution of the copper tartaric complex by the addition of formaldehyde as a reducing agent (Figure 1). Adhesive aluminum strips were attached to the copper-coated textile to manufacture a Cu–Al thermocouple. Six aluminum strips were attached to the same copper textile independently, so that a total of five measuring terminals and a common reference point were formed. A thermal image of the setup is given in the supplementary information (Supplementary Figure S1).

**Figure 2:**
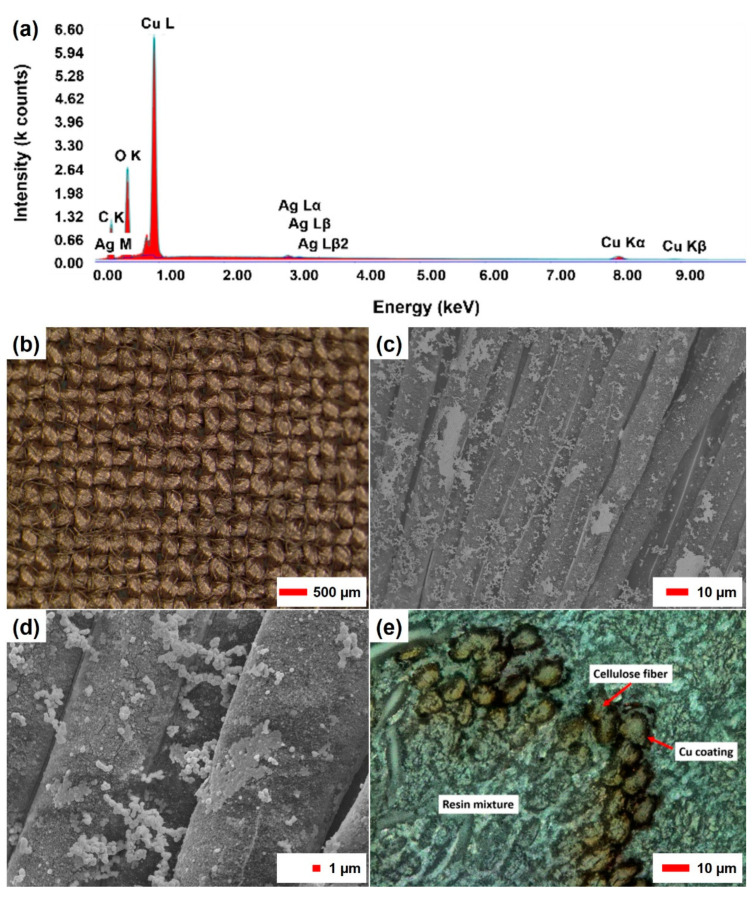
Characterization of the surface of copper-coated lyocell fabrics. (**a**) Shows the result of EDX measurements showing large amounts of copper deposited on the fiber material, (**b**) optical microscopy shows uniform copper deposition, and (**c**,**d**) scanning electron microscopy shows the growth of different formations on the surface. Image (**e**) shows a laser scanning microscopy image of a cross-section, which indicates that almost 50% of the fibers of one yarn are coated with copper.

In a second approach, a series of thermocouples was arranged as aluminum/copper couples to form a thermoelectric generator. Three aluminum/copper joints were heated, while the others were kept at −1 °C (Figure 2).

Although copper–aluminum thermocouples do not exhibit significant differences in the Seebeck coefficients (Table 1), and are therefore not particularly recommended for use, this combination was chosen on purpose. Nickel or nickel-containing compounds such as constantan or nichrome show significantly better values, especially with regard to the figure of merit *Z* (Equation (1)). However, these must be used with caution due to the stimulation of allergic reactions. The figure of merit is calculated from the Seebeck coefficient *S*, the electrical conductivity *σ*, and the thermal conductivity *κ*, and serves as a guide for material combinations in order to achieve a higher thermoelectric conversion efficiency [13].

(1) $$Z=\frac{S^{2}\;\sigma }{\kappa }$$

Hence, materials such as platinum, aluminum, copper, gold, silver and iron are the best options for constructing thermocouples intended for use near human skin. For the figure of merit, changes in the electrical and thermal conductivity also have to be taken into account, which result from the use of a copper coating and a metal film or thread instead of bulk materials.

In a simplified approach, relative Seebeck coefficients *S_AB_* of the selected material combination can be calculated according to Equation (2). The *S_AB_* value is three for the aluminum–copper thermocouples. In terms of quality and skin compatibility, the materials used can be regarded as the least questionable material combinations alongside organic thermoelectric materials.

(2) $$S_{AB}=S_{B}-S_{A}=\frac{\Delta V_{B}}{\Delta T}-\frac{\Delta V_{A}}{\Delta T}$$

### 3.2. Characterization of the Copper-Coated Fabric

An analytical determination of the metal content on the coated fabric using AAS indicated a Cu content of 147 mg/g samples. This corresponds to a total copper content of 24.83 g/m^2^ of the copper-coated fabric. The Ag content was determined with a 6.5 mg/g sample (1.09 g/m^2^ Ag), which indicates a high efficiency of the silver seed for the copper deposition. EDX measurements (Figure 2a) performed on the surface of single fibers confirm these values, as large amounts of copper, including elemental copper and copper oxides, as well as significantly lower amounts of silver and carbon, have been detected on the surface of the copper-coated textile. The morphology of the copper deposit was analyzed using light microscopy, SEM, and LSM, as shown in Figure 2b–e. Using optical microscopy (Figure 2b), the lyocell substrate shows a uniform copper coating, with the structure of the textile remaining visible. This indicates the coating of individual fibers instead of the formation of a film-like layer covering the entire textile. SEM images (Figure 2c,d) taken at individual spots confirm this assumption and show the formation of several different copper structures, including (1) a uniform sheet-like base layer that covers the entire surface of individual fibers, (2) island-like structures that look like incomplete layers, and (3) variable formations of copper particles scattered all over the surface. The presence of different copper aggregates on the surface indicates that on top of the conductive copper layer, additional deposition of particulate material has occurred. A laser microscope image (Figure 2e) of the cross-section of a yarn in the fabric shows that only the outer fibers of the yarn have been coated, and the fibers present in the core of the yarn remain uncoated.

This can be explained with limited chemical exchange and diffusional transport into the core of the yarn. Based on these observations, the layer thickness of the coating was calculated on the basis of the total mass of the metal deposits (Cu and Ag) and from an estimation of the accessible surface of the fabric used. Based on the number of coated fibers visible in Figure 2e and the total number of fibers in the yarn cross-section, the share of fibers accessible can be estimated as about 50%. For the fibers inside the yarn structure, limitations with regard to accessibility and exchange of reagents prevent the formation of deposits. The electroless copper deposition thus proceeds only on the fibers that are near to the fabric surface. The proportion of accessible and coated fibers in a yarn is therefore assumed to be 50%, which is included in the following calculation as a deposition factor (*df*) of 0.5. The factor *df* describes the share of total fiber surface, which is available for copper deposition in a textile structure. If the fabrics are considered as a double-sided coated flat foil, the total layer thicknesses can be estimated to be 1.44 µm based on Equation (3), which describes the theoretical maximum thickness of the plain copper layer.

(3) $$d_{Coating}=\frac{m_{Copper}}{2\;A_{Textile}\;\rho_{Copper}}+\frac{m_{Silver}}{2\;A_{Textile}\;\rho_{Silver}}$$

However, this calculation does not take the entire surface area of the fiber material into account, thus the result of this calculation only gives a rough value that can be considered as an upper limit. In order to present a more realistic coating thickness, the total surface area of the textile has to be taken in account, which is calculated from the available data stated in the materials section, including the mass per area of the fabric *m_Fabric_*_(cm^2^)_ = 0.0143 g/cm^2^, the linear density of the fibers *λ_m_*_(*Fibers*)_, and the density of lyocell *ρ_Lyocell_* = 1.5 g/cm^3^. To determine the fiber surface, first their total length in 1 cm^2^ fabric has to be calculated, as shown in Equation (4).

(4) $$L_{Fibers\left({\mathrm{cm}}^{2}\right)}=\frac{V_{Fabric}}{V_{Lyocell}}=\frac{m_{Fabric\left({\mathrm{cm}}^{2}\right)}\;\rho_{Lyocell}}{\rho_{Lyocell}\;\lambda_{m\left(Fibers\right)}}=\frac{m_{Fabric\left({\mathrm{cm}}^{2}\right)}}{\lambda_{m\left(Fibers\right)}}$$

The surface area of the fibers in 1 cm^2^ can then be determined from the mean cross-section of the fibers and the previously calculated length using Equation (5).

(5) $$A_{Fibers\left({\mathrm{cm}}^{2}\right)}=\pi \;L_{Fibers}\;d_{fibers}=2\pi \;L_{Fibers}\;\sqrt{\frac{V_{Fabric}}{\pi \;L_{Fibers}}}$$

The combination of Equations (4) and (5) leads to a generally applicable formula for calculating the total fiber surface area from the fabrics mass, the linear density of the fibers, and the density of the fiber material used, which is shown in Equation (6).

(6) $$A_{Fibers\left({\mathrm{cm}}^{2}\right)}=2\pi \;m_{Fabric\left({\mathrm{cm}}^{2}\right)}\;\sqrt{\frac{1}{\pi \;\lambda_{m\left(Fibers\right)}\;\rho_{Lyocell}}}$$

The thickness of the coating can then be calculated by replacing the measured surface of the textile 2 *A_Textile_* by the term (*A_Textile_ A_Fibers_*) to calculate with the total surface area given by the fibers, as shown in Equation (7).

(7) $$d_{Coating}=\frac{m_{Copper}}{\rho_{Copper}\;A_{Textile}\;A_{Fibers}}+\frac{m_{Silver}}{\rho_{Silver}\;A_{Textile}\;A_{Fibers}}$$

Using Equation (7), the minimum sheet thickness of the coating is determined to be 79.2 nm. It is worth noting that this formula considers the coating of all fibers, and thus corresponds to a degree of coverage of 100%. However, based on the cross-sectional image in Figure 2e the share of coated fibers can be estimated as 50%. In addition, a lower coverage at the yarn intersections in the fabric has to be considered [11]. Thus, half the coverage ratio at the intersections is assumed. Based on the number of intersection points and the space in between, the intersection points are estimated to represent 80% of the fabric surface, and the yarns represent 20% of the fabric surface, which further limits the effective area available for copper deposition. Taking a deposition factor of 0.25 for 80% of the surface and a deposition factor of 0.5 for 20% of the more accessible yarn surface into account, an average deposition factor of 0.3 can be calculated for the entire textile sample. Based on these considerations, the resulting coating-layer thickness is estimated to be approximately 257 nm. The complete calculation is shown in Supplementary Figure S2.

The SEM and EDX results, in combination with AAS, yield a basis for an estimation for an average thickness of the coating, which, however, must be understood as a simplified model calculation to generate an average measure for the copper layer thickness in the textile.

### 3.3. Characterization of Textile-Based Thermocouples

**Figure 3:**
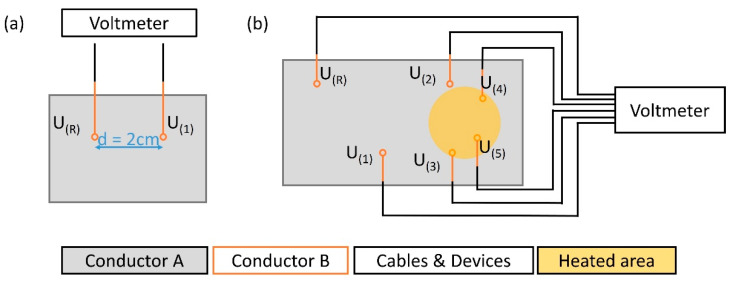
Measurement arrangement used for characterization of the (**a**) flexible thermal element and (**b**) the multi-point temperature sensor array using five measuring terminals and one reference terminal connected to the voltmeter.

The use of copper-coated fabrics enables the construction of flexible thermocouple sensor arrays that are suitable for multi-channel temperature measurements and temperature mapping. The thermocouples presented in this work are constructed as pairs of thermocouples. The setup consists of a common copper-coated fabric (conductor A), which is in contact with independent aluminum strips (conductor B) that form a thermal element (Figure 3a). The thermocouple array consists of five temperature measurement terminals (U_(1)_–U_(5)_) and a reference terminal (U_(R)_), which is kept at a constant temperature (Figure 3b), thus five independent thermal elements are formed on the copper-coated fabric. By keeping the reference terminal U_(0)_ at a fixed temperature while applying heat to the measurement terminals U_(1)_–U_(5)_, a thermoelectric signal is generated in the form of an output voltage that depends on the material of the conductors and the temperature difference between the measurement terminals and the reference terminal.

**Figure 4:**
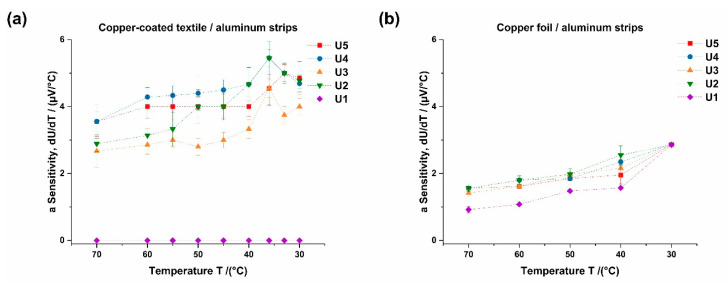
Temperature measurements performed using two different setups consisting of five measuring terminals and one reference terminal placed on (**a**) a copper-coated lyocell fabric, and (**b**) a copper foil. The dashed lines between the measured points were added to guide the eye.

The assemblies used for the temperature measurements consisted of a copper-coated cellulose fabric with a size of 44 cm^2^ and six aluminum strips (1 cm width and 4 cm length). The contact area between each strip and the surface of the copper-coated cellulose fabric was 1.0 cm^2^. Five different thermoelectric signals between the measurement terminals (U_(1)_–U_(5)_) and the reference terminal were recorded within a temperature range of 70 °C to 27 °C. A beaker filled with hot water served as the heat source for the measurement (Figure S1). The thermoelectric coefficient *dU/dT* was calculated from the voltage difference registered during the cooling, and the respective temperature difference between the measurement terminals and the reference terminal. In a temperature range from 70 °C to 40 °C, the temperature measurements led to an almost constant thermoelectric coefficient for the measurement terminals U_(2)_ to U_(5)_. Measurement terminal U_(1)_ was not directly heated by the heat source and remained at ambient temperature during the experiment. The low thermal conductivity of the sensor textile prevented heat transfer along the copper-coated layer, from the heated area to the position of U_(1)_. Thus, no signal was generated that would allow the calculation of a thermoelectric coefficient, as shown in Figure 4a.

Depending on the respective measurement terminal and the heat transferred to the specific measurement spot, the thermal elements based on copper-coated textiles (Figure 4a) show average output voltages between 2.8 µV/°C and 4.4 µV/°C in a temperature range between 70 °C and 40 °C. Since the reference temperature corresponded to the ambient temperature of 27 °C, the measurement inaccuracies became more relevant for the thermoelectric output voltages between 40 °C and 27 °C, which is reflected in the calculated values for dU/dT. These results are comparable to the measurements with Cu/Al wires reported by Kharote et al. [14]. The small difference in the Seebeck coefficients of copper and aluminum lead to the comparatively low temperature sensitivity of the thermocouple. However, for applications that are intended to be used near the skin, copper–aluminum-based concepts are favorable compared to setups using nickel or constantan, which can corrode and cause allergies and skin irritation. For comparison, a setup using the same geometries made from copper and aluminum foils (Figure 4b) was built. This setup serves as a proof-of-concept for the manufacture, and as a reference system with the use of metal foils as conductors. Due to the higher thermal conductivity along the copper foil, heat transfer from the measurement terminals U_(2)_–U_(5)_ towards measurement terminal U_(1)_ and the reference terminal U_(0)_ is observed. This leads to a lower temperature difference between the measuring terminals and the reference terminal, as well as to the apparent decrease in the thermoelectric coefficients dU/dT. Compared to the metal film systems, the advantage of sensor systems that use thin copper coating on flexible substrates, is that a significantly lower heat flow along the metal layer leads to a higher spatial resolution of the thermal element matrix. The higher spatial resolution is thus attributed to the lower thermal conductivity, which depends on the layer thickness (300 W/m K at 250 nm sheet thickness) as well as on the quality of the conductor [15]. With thick copper films, only larger distances between the measuring terminals provide reliable temperature information, since the heat flow between the measuring terminals must be minimized.

**Figure 5:**
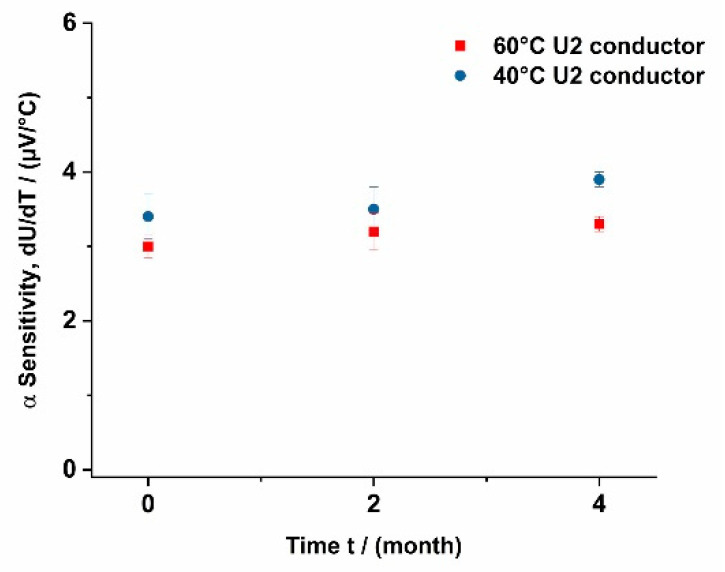
Long-term stability of sensor arrangements using copper-coated lyocell fabrics as substrates.

In addition to the overall functionality, aging effects also play an important role for an assessment of the durability in use. Therefore, the long-term stability of the conductive copper matrix and the sensor arrangement was also examined. Visually, no significant differences between the new and aged sensor textiles could be observed using optical and laser scanning microscopy. The measurement of the thermoelectric coefficient also showed only minor dependency on the storage time. During a storage of four months at ambient temperature, an increase in the thermoelectric coefficient from initially 3 × 10^−3^ mV/K to 4 × 10^−3^ mV/K is observed. The slight increase in thermoelectric coefficient during a storage period of four months remains within the statistical error of the measurements and remains within the range of the results shown in Figure 5.

Compared to recently published results on the manufacture of temperature sensors for e-textiles, the presented system exhibits a rather low sensitivity, which is mainly due to the use of copper–aluminum thermocouples, as shown in Table 2. Due to the poor comparability with systems with other functional principles, a comparison with resistance-based, optical and other systems is dispensed with at this point.

### 3.4. Characterization of Thermoelectric Generator

**Figure 6:**
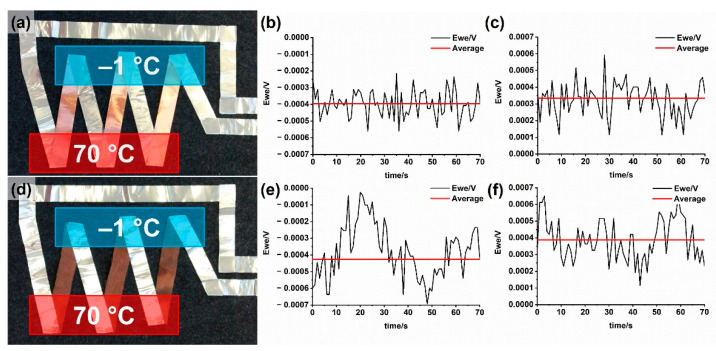
Thermoelectric output voltages of arrangements in series of three thermocouples using strips of copper and aluminum foils (**a**–**c**), and strips of copper-coated textiles and aluminum foils (**d**–**f**). Temperature difference 71 K, (**b**) Cu-strips + Al-strips, (**c**) same arrangement with reverted temperature gradient, (**e**) Cu-coated textile + Al-strips, (**f**) same arrangement with reverted temperature gradient.

The thermoelectric output voltage of both of the assemblies (Figure 6) is comparable. An average voltage of 0.4 mV was recorded for a temperature difference of 71 K between the junctions. A lower internal resistance of the copper-foil-based assembly was measured with 40 Ohms, while the replacement of the copper strips by copper-coated textile strips increased the internal resistance to 708 Ohm. As the resistance of the metal foils is rather low (aluminum 7 cm × 1 cm, 0.3 Ohms; copper 7 cm × 1 cm, 0.1 Ohms), the major contribution to the overall internal resistance results from the contact points in the junction. The higher resistance of the copper-textile-based arrangement results from the higher resistance of the copper-coated textile strips (7 cm × 1 cm, 72 Ohms) and the contact resistance at the junctions. Based on the internal resistance (*R_i_*), the load resistance (*R_L_*), and the thermoelectric output voltage (*U*), the power of the thermoelectric generator can be estimated (Equation (8)).

(8) $$P=\frac{U^{2}\;R_{L}}{{\left(R_{L}+R_{i}\right)}^{2}}$$

For an electrical consumer with a resistance of 1000 Ohm, the result is a current flow of 234 nA if the internal resistance of the assembly is considered with 708 Ohm. In this case, a power output of 5.5·10^−11^ Watt can be realized at the consumer. An optimization of the assembly with an increased number of junctions, an optimized geometry and the use of copper-coated textiles that show even lower electrical resistivity [20], will directly lead to a higher energy output. This becomes clear when comparing the textile-foil-based assembly with the foil-based assembly, which offers a current flow of 385 nA and a power output of 14.7·10^−11^ Watt.

A comparison with other systems shows that most systems suffer from either integration problems, abrasion resistance or even only high costs (Table 3). A direct comparison of the system presented is hardly possible due to the small number of thermocouples, but extrapolating the area used would lead to a low-to-moderate result in output power.

## 4. Conclusions

The electroless deposition of copper layers on cellulose fabrics enables the construction of a flexible thermoelectric sensor matrix. Using aluminum as the second material, a thermoelectric coefficient of 3 to 4 µV/K was realized, which is sufficient to manufacture textile-based temperature measurement devices. The model calculations for estimating the thickness of the copper layer on the fabric structure resulted in an average thickness of approximately 260 nm. The thermoelectric coefficients are comparable to values obtained using similar setups with copper films with a thickness of 0.1 mm. The conductivity of the copper layer with 16.5 Ohm/square [11] was sufficient to achieve signal transfer, but the thermal conductivity of the metal coating is still significantly lower compared to a copper foil with 50 µm. During a storage time of four months, no significant change in thermoelectric coefficient was observed, indicating no aging such as corrosion of the surface.

A demonstrator for a flexible thermoelectric energy generator was successfully manufactured and tested. A combination of three thermocouples connected in a series led to an output voltage of 0.4 mV at a temperature difference of 71 K, which is comparable to a copper-foil-based reference setup.

The examples given demonstrate the potential of flexible thin-film-coated textiles as materials for the construction of sensors and their integration into garments to form functional e-textiles.

## Figures and Tables

**Table 1:** Calculation of the figure of merit, using the given parameters.

Material	*S* Seebeck Coefficient (µV/K)	*σ* Electrical Conductivity (S/m)	*κ* Thermal Conductivity (W/m*K)	*Z* Figure of Merit (1/K)
Constantan	−40	20.4	21.2	1539.6
Nickel	−20	143.0	90.9	629.3
Platinum	−5	94.3	71.6	32.9
Aluminum	−1.5	377.0	237.0	3.6
Copper	1.5	596.0	401.0	3.3
Gold	1.5	411.0	318.0	2.9
Silver	1.5	630.0	429.0	3.3
Iron	13	100.0	40.4	418.3
Nichrome	20	6.7	11.3	237.2

**Table 2:** Comparison to relevant literature on the manufacture of textile-based thermocouples.

Materials Used	Technique of Incorporation into Textiles	Comments	Sensitivity (µV/°C)	References
Steel knitted fabric/Constantan wire	Glued connections on steel knitted fabric	Low flexibility, high sensitivity, high abrasion resistance, allergenic substance	41.8	[16]
PEDOT:PSS/PANI	Screen printed	High flexibility, moderate sensitivity, low abrasion resistance	10	[17]
Carbon yarn/nickel-coated carbon yarn	Proposed stitching	Moderate flexibility, high sensitivity, moderate abrasion resistance, allergenic substance	145 (10 pairs)	[18]
	Stitched thermopiles		11.61 (1 pair)	[19]

**Table 3:** Overview of different energy harvesting devices, designed to be integrated into textiles.

Operational Technique	Materials Used	Technique of Incorporation in Textiles	Comments	Output Power	References
Piezoelectric	ZnO nanowire coated aramid fibers	Proposed weaving	High energy output, high cost, low abrasion resistance, moderate flexibility	20–80 W/m^2^	[21]
Thermoelectric	Conventional thermoelectric module	Glued	High energy output, low cost, high abrasion resistance, low flexibility	0.8 to 1 mW	[22,23]
Thermoelectric	PEDOT:PSS coated silk thread/silver-plated polyamide thread	Stitched	Moderate energy output, moderate abrasion resistance, moderate flexibility	1.2 µW at Δ*T* = 65 °C 0.2 µW at Δ*T* = 65 °C	[24]
Thermoelectric	Antimony and bismuth printed on capton	Attached as a coil	High energy output, high abrasion resistance, low flexibility	2 µW at Δ*T* = 5 °C	[25]
Photovoltaic	PP fiber coated with PEDOT:PSS, P3HT:PCBM, and LiF/Al	Proposed weaving	High energy output at ambient light, moderate flexibility, high cost	0.11 W	[26]

## Data Availability

The data that support the findings of this study are available from the corresponding author, upon reasonable request.

## Supplementary Materials

The following are available online at https://www.mdpi.com/article/10.3390/s21113742/s1, Figure S1: thermal image of the thermocouple array, Figure S2: calculation of copper layer thickness.
