# Supplementary material for: Multi-Point Flexible Temperature Sensor Array and Thermoelectric Generator Made from Copper-Coated Textiles
Source: Sensors (Basel). 2021 May 28;21(11):3742. doi: 10.3390/s21113742 (PMC8198943; doi:10.3390/s21113742)
Supplement: Supplementary file 1 [file sensors-21-03742-s001.zip › sensors-1210100-supplementary.pdf]

Supplementary Materials

Multi point flexible temperature sensor array and thermoelectric generator made from copper-coated textiles

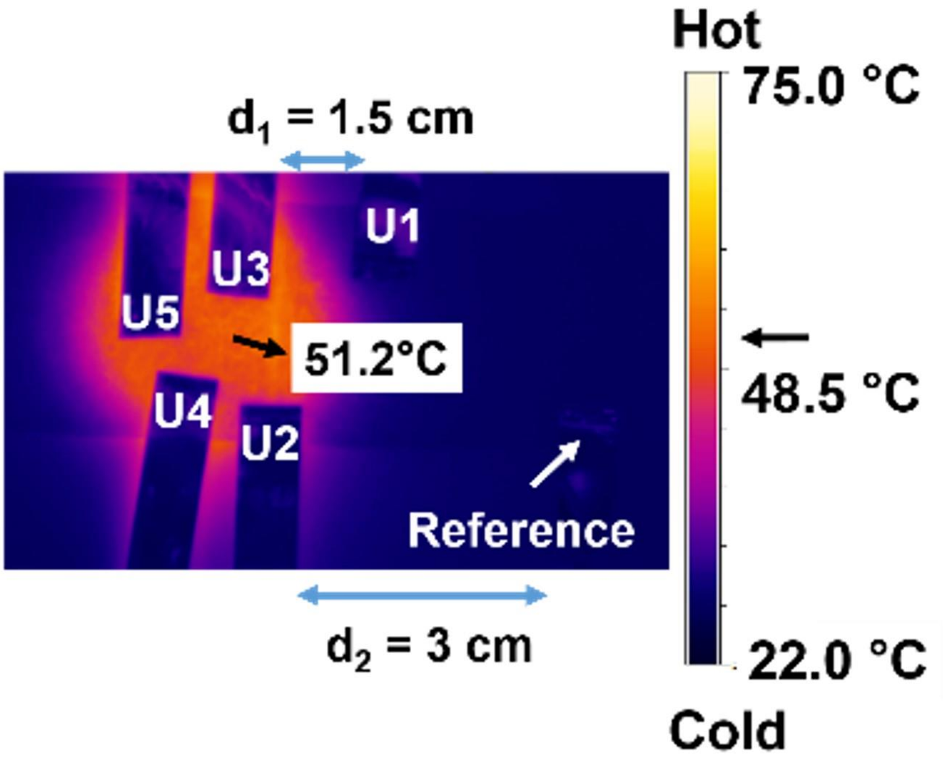

Figure S1. Thermal image of the measurement arrangement of the thermo-sensor.

Coating details (calculation as a continuous film)

Copper content	Silver content	Coating total	Sample total	Textile weight	Sample size
147 mg/g	6.5 mg/g	153.5 mg/g sample	0.8465 mg	143 g/10,000 cm²	59.2 cm²

Copper density	coating thickness total (film)
8.96 g/cm³	0.000277 cm
	2.771522 µm

Fabric details

31 threads per cm   62 threads per cm?   ?

100 fibers per thread ?

Fiber diameter	Fiber surface per 1 cm	surface of 1 yarn per 1 cm
10 µm	0.003142 cm²	0.314159265 cm²

Consideration:

0.5 coating ratio in spots w/o crossing points   0.33 ratio of fabric size

0.25 coating ratio in spots showing crossing points   0.67 ratio of fabric size

Total coated surface area of fabric	Coating thickness under these considerations
6.476393 cm²	0.428 µm

0.142	µm if all fibers are covered compleetely
0.285	µm if 50% of the total fibers are covered compleetely
0.428	µm if 50% of single yarns are coated and 25% of fibers at crossing points are coated
0.517	µm if the crossingpoint ratio is 9/1
0.569	µm if there are only crossing points

Figure S2. Calculation of the copper layer thickness under consideration of differently coated areas all over the fabric. The calculation also takes the fiber fineness and the deposited amounts of copper and silver into account.
